# Prevalence of potential sports‐associated risk factors in Swiss amyotrophic lateral sclerosis patients

**DOI:** 10.1002/brb3.630

**Published:** 2017-03-16

**Authors:** Nina Feddermann‐Demont, Astrid Junge, Konrad P. Weber, Michael Weller, Jiří Dvořák, Alexander A. Tarnutzer

**Affiliations:** ^1^ Department of NeurologyUniversity Hospital Zurich and University of ZurichZurichSwitzerland; ^2^ Swiss Concussion CenterSchulthess ClinicZurichSwitzerland; ^3^ FIFA Medical Assessment and Research Centre (F‐MARC)ZurichSwitzerland; ^4^ Schulthess ClinicZurichSwitzerland; ^5^ Medical School Hamburg (MSH)HamburgGermany; ^6^ Department of OphthalmologyUniversity Hospital Zurich and University of ZurichZurichSwitzerland; ^7^ Fédération Internationale de Football AssociationZurichSwitzerland

**Keywords:** amyotrophic lateral sclerosis, football (soccer), head trauma, ice hockey, physical activity

## Abstract

**Objectives:**

Amyotrophic lateral sclerosis (ALS) has been reported to occur with increased incidence amongst physically active people. The role of extrinsic risk factors as physical activity, head trauma and drug/pesticide‐exposure in the pathophysiology of ALS and especially in the context of practising sports remains controversial.

**Materials and Methods:**

We retrospectively studied exposure to extrinsic factors in 92 ALS patients in the presymptomatic stage. Metabolic equivalents (METs) were calculated and the association of physical activity, drug intake, head trauma and participation in specific sports (football [soccer], ice hockey) with age at symptom‐onset was evaluated.

**Results:**

Ninety‐five percent of patients considered themselves physically active before symptom‐onset. Total MET‐values varied broadly and there was no correlation between vigorous physical activity and age at symptom‐onset. Mild traumatic brain injury (prevalence = 16.7%) was the most frequent diagnosis after head/neck injury. A history of ≥1 head/neck injuries was associated with a younger age at symptom‐onset (61.8 ± 11.0 vs. 54.1 ± 13.0, *p* = .013). In former football and ice hockey players the rate of vigorous physical activities was increased (*p* < .05), whereas total MET‐values, frequency of head injuries and analgesic intake were not different compared to other ALS patients.

**Conclusions:**

History of head injuries was the only extrinsic risk factor associated with accelerated neurodegeneration in ALS. There was no evidence for extrinsic factors predisposing former football and ice hockey players to ALS. Our data therefore support the hypothesis that not increased physical activity per se, but other unknown environmental factors and/or genetic profile or lifestyle‐promoting physical fitness increases ALS susceptibility.

## 1 Introduction

Progressive neurodegeneration of upper and lower motor neurons is the hallmark of amyotrophic lateral sclerosis (ALS, also known as “Lou Gehrig disease”), which remains poorly understood in its pathophysiology and lacks effective treatment options (Kiernan et al., 2011). The incidence of ALS in Western countries is between 1–3/100,000 per person years (Alonso, Logroscino, Jick, & Hernan, 2009; Beghi et al., 2007; Traynor et al., 1999) with men affected more frequently than women (male‐to‐female rate between 0.8:1 and 2.7:1; Alonso et al., 2009; Cronin, Hardiman, & Traynor, 2007; Uenal et al., 2014). Only 10%–15% of ALS cases are familial, while the remaining 85%–90% are considered sporadic (Kiernan et al., 2011). In these cases, likely both genetic and environmental (extrinsic) factors contribute to the aetiology of ALS (Harwood, Mcdermott, & Shaw, 2009). Mean (or median) age at symptom onset for sporadic ALS ranges between 63 and 67 years (Logroscino et al., 2010; Piemonte and Valle d'Aosta Register for Amyotrophic Lateral Sclerosis, 2001; Uenal et al., 2014).

In the past, reported clusters of ALS in professional football (soccer) players in Italy (Belli & Vanacore, 2005; Chio, Benzi, Dossena, Mutani, & Mora, 2005) and American football players in the US (Lehman, Hein, Baron, & Gersic, 2012) have received intense media coverage and have fuelled the controversy about the significance of sports‐related extrinsic risk factors in ALS (Beghi, 2013). Several potential explanations were brought forward for excess occurrence of ALS in former football players: (1) vigorous physical activity; (2) football‐specific trauma and heading‐related microtrauma; (3) illegal toxic substances or chronic misuse of (anti‐inflammatory) drugs; and (4) exposure to pesticides (Chio et al., 2005; Vanacore, Binazzi, Bottazzi, & Belli, 2006).

While head injuries were linked to increased risk for ALS in some studies (Chen, Richard, Sandler, Umbach, & Kamel, 2007; Pupillo et al., 2012; Strickland, Smith, Dolliff, Goldman, & Roelofs, 1996), others observed a significant association only within 1 year after head injury (Peters et al., 2013; Turner, Abisgold, Yeates, Talbot, & Goldacre, 2010), rather suggesting accidents provoked by early signs of the disease. Some studies report that (vigorous) physical activities have been associated with an increased risk for ALS (Beghi et al., 2010; Gotkine, Friedlander, & Hochner, 2014), while others did not find any excess risk (Huisman et al., 2013; Longstreth, Mcguire, Koepsell, Wang, & van Belle, 1998; Veldink et al., 2005) or even reported a protective effect (Pupillo et al., 2014). Toxins like herbicides, pesticides, and fertilizers were linked to ALS (Al‐Chalabi & Leigh, 2005), and the intake of nonsteroidal anti‐inflammatory drugs was associated with a twofold increased risk for ALS in men in one study (Popat et al., 2007), while this was not confirmed by others (Fondell et al., 2012).

Overall, the role of physical activity, head trauma and drug/pesticide exposure in the pathophysiology of ALS, and especially in the context of participation in specific sports remains controversial. Taking into consideration that recreational sports activities are very popular, exposure to such potential risk factors may have far‐reaching effects on a large scale.

We therefore systematically collected data on presymptomatic head injuries, physical activities and exposure to other proposed risk factors in a cohort of Swiss ALS patients. A special focus was put on the popular contact and collision sports football and ice hockey linked to vigorous physical activity and augmented risk for head trauma. We investigated the hypothesis that specific sports activities predispose for ALS. If this hypothesis is true, the physical activity profile in former players is expected to be higher than in other ALS patients, and first symptoms are predicted to occur earlier in life. Likewise, higher rates of head injuries and earlier disease‐onset in ALS patients that previously participated in one (or more) of these sports are predicted.

## 2 Material and Methods

The study protocol was approved by the Cantonal ethics commissions Zurich and Aargau and was in accordance with ethical standards laid down in the 2013 Declaration of Helsinki for research involving human subjects. All participants gave written informed consent for participation in the study.

### 2.1 Data collection

Using the registry of the Swiss Federation for Joint Tasks of Health Insurers (SVK) that was set‐up to handle requests for riluzole, all 175 ALS patients that had submitted requests in 2011 were contacted by the SVK via regular mail, and invited to answer a questionnaire anonymously with 44 questions covering disease onset and current symptoms (including the ALS functional rating scale) as well as head injuries, intake of pharmaceuticals and physical activities in the presymptomatic stage in general and specifically regarding participation in football and/or ice hockey. Diagnosis of ALS was confirmed by the participating patients, but was also previously assessed by the registry. Specifically, the SVK has designed a standardized registration form that systematically asks about ALS‐related findings in order to distinguish between patients that meet accepted diagnostic criteria for ALS (involvement of upper and lower motor neurons in at least two regions) and ALS mimics not eligible for riluzole. This evaluation is applied for all requests submitted to the registry.

### 2.2 Calculation of intensity of physical activity

Metabolic equivalent (MET) values were calculated based on reported physical activities and their corresponding MET values (Metabolic Equivalent (MET) Values for Activities in American Time Use Survey (ATUS)). Total MET values were determined by multiplying the MET value, the duration in years and the hours per week spent for this activity (see also (Huisman et al., 2013) for details). Vigorous physical activities were defined as activities with a MET >6.

### 2.3 Data processing and statistical analysis

Data were collected and processed in Excel 2010 (Microsoft Corporation, Redmond, WA, USA). Analysis of variance (ANOVA) and odds ratios (OR) were used for statistical analysis (SPSS Version 22).

## 3 Results

### 3.1 General findings

Response rate for questionnaires was 52.6% (92/175). For demographics including age, gender, and disease duration see Table 1. Impairments were most pronounced for walking and writing abilities. A gender difference for age at symptom‐onset (*p* = .033) and for age at diagnosis (*p* = .019) was observed, with ages being lower for men than for women (56.8 ± 12.2 vs. 62.1 ± 10.8 years).

**Table 1:** Epidemiological data of the amyotrophic lateral sclerosis (ALS) cohort

	Total *n* = 92 Mean (1 *SD*)	Men *n* = 51 (55.4%) Mean (1 *SD*)	Women *n* = 41 (44.6%) Mean (1 *SD*)	Δ (m vs. w); *p* (ANOVA)
Age (years) at:				
First symptoms	59.2 (11.9)	56.8 (12.2)	62.1 (10.8)	4.71; **.033**
Diagnosis	60.3 (11.7)	57.7 (11.9)	63.5 (10.8)	5.75; **.019**
Time of evaluation	65.7 (11.6)	63.7 (12.1)	68.3 (10.5)	3.65; .059
Delay of diagnosis (months)	16.8 (15.8)	17.1 (15.2)	16.4 (16.6)	0.04; .837
Months since diagnosis	60.6 (50.7)	63.7 (48.6)	56.9 (53.5)	0.41; .523
BMI before illness (kg/m^2^)	25.3 (3.5)	25.7 (3.0)	24.8 (4.0)	1.35; .249
Difficulties to^a^:				
Write^b^	2.00 (1.5)	1.72 (1.5)	2.34 (1.4)	3.89; .052
Speak^c^	2.57 (1.6)	2.98 (1.4)	2.07 (1.8)	7.44; **.008**
Walk^d^	1.76 (1.3)	1.82 (1.3)	1.68 (1.3)	0.24; .625
Breath^e^	2.87 (1.4)	2.76 (1.4)	3.00 (1.3)	0.71; .402
Number of activities (*n*)	3.0 (1.6)	3.2 (1.6)	2.8 (1.6)	1.08; .302
MET of activities (×10^6^)	5.1 (5.8)	4.9 (6.8)	5.2 (4.3)	0.09, .759
Total MET values (×10^6^)	13.9 (12.9)	12.2 (11.0)	16.1 (14.8)	1.99; .162
Physical activities	*n* (%)	*n* (%)	*n* (%)	χ^2^; *p*
Sports	76 (82.6)	44 (86.3)	32 (78.0)	1.07; .301
Domestic work and family care	49 (53.3)	18 (35.3)	31 (75.6)	14.84; **<.001**
Job‐related activities	46 (50.0)	23 (45.1)	23 (56.1)	1.10; .294
Others^f^	10 (10.9)	3 (5.9)	7 (17.1)	2.94; .087
History of head injuries				
1	10 (10.9)	5 (9.8)	5 (12.2)	4.57; .471
2 or more	12 (13.0)	9 (17.6)	3 (7.3)	

a Self‐ratings of ALS patients.

b According to ALS Functional rating scale (ALS‐FRS): 4—Normal, 3—Slow or sloppy; all words are legible, 2—Not all words are legible, 1—Able to grip pen but unable to write, 0—Unable to grip pen.

c According to ALS.FRS: 4—Normal speech processes, 3—Detectable speech disturbance, 2—Intelligible with repeating, 1—Speech combined with nonvocal communication, 0—Loss of useful speech.

d According to ALS‐FRS: 4—Normal, 3—Early ambulation difficulties, 2—Walks with assistance, 1—Non‐ambulatory functional movement only, 0—No purposeful leg movement.

e According to ALS‐FRS: 4—None, 3—Occurs when walking, 2—Occurs with one or more of the following: eating, bathing, dressing (ADL), 1—Occurs at rest, difficulty breathing when either sitting or lying, 0—Significant difficulty, considering using mechanical respiratory support.

f Volunteer work, assistance work, to cut down trees, dog sports, painting, travelling, collecting on jumble sales, sauna, and handcraft work.

Statistically significant p‐values are in bold.

### 3.2 Physical activity profiles before symptom‐onset

**Figure 1:**
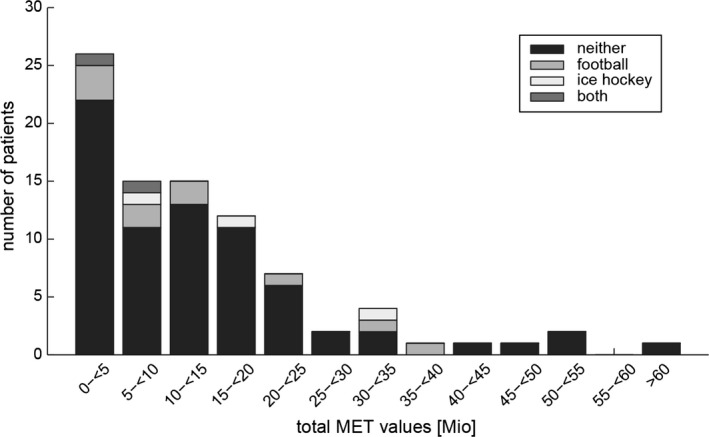
Distribution of total individual metabolic equivalent values (in bins of five millions) plotted against the number of amyotrophic lateral sclerosis patients (*n* = 92) falling into these bins. Four subgroups are distinguished, based on former participation in football, ice hockey, both sports, or neither of these sports

Ninety‐five percent of patients considered themselves physically active before symptom‐onset. The number of physical activities (Table 2a) and individual total MET values varied considerably between patients (Figure 1). The most frequently reported sports‐related physical activities were endurance sports (biking/running in 42.4%; climbing/hiking in 30.4%). Vigorous physical activities were identified in 51.1% of patients. Higher total MET values were correlated with symptom‐onset later in life (*n* = 91, missing value in 1 subject, Pearson correlation = .201, *p* = .032), while there was no correlation (*p* = .247) between vigorous physical activity and age at symptom‐onset.

**Table 2:** (a) Physical activities of amyotrophic lateral sclerosis patients before symptom onset, (b) head/neck injuries leading to medical evaluation or being accompanied by very intense complaints

(a)	Total (*n* = 92)	%	Men (*n* = 51), *n* (%)	Women (n = 41), *n* (%)	
Physically active before symptom onset	86	94.5	47 (92.2)	39 (97.5)	
Physical activities^a^					
Sports	76	82.6	44 (86.3)	32 (78.0)	
Biking/running	39	42.4	28 (54.9)	11 (26.8)	
Hiking/climbing	28	30.4	17 (33.3)	11 (26.8)	
Dance/fitness/gymnastics	25	27.2	12 (23.5)	13 (31.7)	
Walking	20	21.7	10 (19.6)	10 (24.4)	
Skiing	14	15.2	9 (17.6)	5 (12.2)	
Hit‐back sports	13	14.1	7 (13.7)	6 (14.6)	
Football	12	13.0	11 (21.6)	1 (2.4)	
Goal sports	6	6.5	5 (9.8)	1 (2.4)	
Swimming	5	5.4	3 (5.9)	2 (4.9)	
Ice hockey	5	9.8	5 (9.8)	0	
Riding	3	3.3	2 (3.9)	1 (2.4)	
Strength training	3	3.3	3 (5.9)	0	
Track and field	2	2.2	2 (3.9)	0	
Other sports^b^	9	9.8	7 (13.2)	2 (4.9)	
Domestic work and family care	49	53.3	18 (35.3)	31 (75.6)	
Domestic work/garden	48	52.2	18 (35.3)	30 (73.2)	
Family care	5	5.4	0	5 (12.2)	
Job‐related activities	46	50.0	23 (45.1)	23 (56.1)	
Music	9	9.8	7 (13.2)	2 (4.9)	
Others^c^	10	10.9	3 (5.9)	7 (17.1)	

Football and ice hockey‐related activities	Age activity started (mean ± 1 *SD*)	Age activity stopped (mean ± 1 *SD*)	Years participated (mean ± 1 *SD*)	Matches per season (mean ± 1 *SD*)	Weekly training (min)
Football	13.2 ± 5.6 years	32.5 ± 17.6 years	19.3 ± 16.4	22.2 ± 5.1	246 ± 219
Ice hockey	20.8 ± 9.4 years	28.6 ± 12.6 years	7.8 ± 5.3	17.5 ± 5.0	113 ± 112

(b) Diagnosis	Number of head/neck injuries in individual patients				
	1	2–3	4–5	>5	Total (%)
mTBI	9	2	1	0	12 (57.1)
Whiplash‐associated disorders	2	0	0	0	2 (9.5)
Abrasion	3	0	0	0	3 (14.3)
Fracture	2	0	0	0	2 (9.5)
Mixed^d^	0	2	0	0	2 (9.5)
Total (%)	16 (22.2)	4 (5.6)	1 (1.4)	0 (0.0)	21/72 (30.0)^e^

mTBI, mild traumatic brain injury.

a Physical activities reported in 87 patients with missing data in the remaining five patients.

b Playing billiard, fishing, inline skating, hunting, sailing, riding a motorcycle, Jiu‐Jitsu, Karate.

c Volunteer work, assistance work, to cut down trees, dog sports, painting, travelling, collecting on jumble sales, sauna, and handcraft work.

d One combination of a fracture and an abrasion, one combination of two fractures and one abrasion.

e Missing values in 20 cases.

### 3.3 Head and neck injuries before symptom‐onset

Information on the presence/absence of previous head/neck injuries was provided by 70 patients (76.1%). More than two‐thirds of these patients (48) (68.6%) reported no head/neck injury that resulted in medical evaluation or was accompanied by very intense complaints (i.e., head/neck pain, dizziness/vertigo, nausea/vomiting, disturbances of vision, impairments in memory/concentration). Out of 22 patients with reported head/neck injury, 12 (54.6%) recalled multiple events. The most frequent diagnosis was mild traumatic brain injury (mTBI, 57.1%; Table 2b). A history of one or more head/neck injuries was associated with younger age at symptom‐onset (*p* = .013), while analyses of number of head/neck injuries and gender did not show any differences (Table 3). The mechanism of head/neck injury was reported in 26/29 events. Most frequent causes were falls during activities of daily life (8/26) and traffic‐related accidents (8/26), followed by sports‐related injuries (4/26) and work‐related injuries (4/26).

**Table 3:** Potential environmental risk factors for amyotrophic lateral sclerosis and their link to age at symptom onset

	Men (*n* = 51, 55.4%) *n* (%)	Women (*n* = 41; 44.6%) *n* (%)	Odds ratio (95% CI)	Age at symptom onset (mean ± 1 *SD*)	*p*‐Value (ANOVA)
Physical activity					
Low (MET < 3)	9 (17.6)	7 (17.1)	1.04 (0.35–3.08)	57.7 ± 13.6	.247
Moderate (3 < MET < 6)	10 (19.6)	5 (12.2)	1.75 (0.55–5.62)		
Vigorous (MET > 6)	32 (62.7)	29 (70.7)	0.70 (0.29–1.68)	60.6 ± 9.9	
Head injury					
No head injury	38 (74.5)	33 (80.5)	1.41 (0.52–3.82)	61.8 ± 11.0	**.013**
≥1 head injuries	13 (25.5)	8 (19.5)		54.1 ± 13.0	
≥1 mTBI	8 (15.7)	5 (12.2)			
Medication^a^					
No	42 (82.4)	31 (75.6)	0.66 (0.24–1.83)	58.1 ± 11.8	.096
Yes	9 (17.6)	10 (24.4)		63.2 ± 11.5	
No NSAIDs	45 (88.2)	36 (87.8)	0.96 (0.27–3.40)	58.8 ± 12.3	.343
NSAIDs	6 (11.8)	5 (12.2)		63.3 ± 4.4	

a Medication included nonsteroidal anti‐inflammatory drugs (NSAIDs; diclofenac [*n* = 6], celecoxib [*n* = 1], ibuprofen [*n* = 3], mefenamid [*n* = 1], rofecoxib [*n* = 1], azapropazone; Kiernan et al., 2011), other analgesics (OPK; paracetamol [*n* = 6], paracetamol plus coffein [*n* = 1[, metamizol [*n* = 2[, tramadol [*n* = 2[) and other drugs (omeprazol [*n* = 2], hydroxichloroquin [*n* = 1], temazepam [*n* = 1], lamotrigin [*n* = 1], cortison [*n* = 1], diazepam [*n* = 1], acetylsalicysäure [*n* = 1], tamsulosin [*n* = 1], enalapril [*n* = 1]).

Statistically significant p‐values are in bold.

### 3.4 Drug intake before symptom onset

Daily drug intake for >30 consecutive days before symptom onset was reported by 19/92 patients (20.7%; Table 3), with analgesics being the most frequently prescribed drugs (16.3%, Table S1). None of the patients (*n* = 89, missing value in three subjects) reported former intake of performance‐enhancing substances such as anabolic steroids.

### 3.5 Subgroup analysis of ALS patients with a history of football and/or ice hockey

Eleven ALS patients participated in football (11/92, 12.0%) before symptom onset, five in ice hockey (5/92, 5.4%) regularly (defined as participating in a club or school team at least once a week for at least 1 year), with two patients playing both sports. Except one female football player, all football and ice hockey players were males, yielding a higher odds ratio of football players amongst men than women (OR = 9.76, 95%‐CI = 1.19–79.77). One additional patient reported football as “other” physical activity, this patient was considered for further analysis as well (Table 2a). Players’ positions were equally distributed for football and ice hockey.

Compared to the total average MET values (in millions) in patients without a history of football or ice hockey (13.9 ± 13.2), values in patients with a history of football (13.9 ± 12.4), ice hockey (18.1 ± 11.9) or both sports (5.8 ± 4.5) were in a similar range (Table S2). ANOVA showed no main effect of former participation in football/ice hockey on total MET values (*F* = 0.361, *p* = .781). Compared to the physical activity with the highest total MET value on an individual basis, MET values resulting from participation in football or ice hockey were moderate, contributing 33.8% (football) and 3.9% (ice hockey), respectively. Vigorous physical activities were identified more frequently in former football/ice hockey players, while the rates of analgesic intake were not different (Table 4).

**Table 4:** former participation in football (FB) and/or ice hockey (IH) and environmental risk factors for amyotrophic lateral sclerosis

	Former FB and/or IH participation (*n*, %)	No FB or IH participation (*n*, %)	Odds ratio (95% CI)
Physical activity (MET value of specific activity)^a^			
Nonvigorous (MET ≤ 6)	3 (20.0)	42 (54.5)	**4.8 (1.3–18.4)**
Vigorous (MET > 6)	12 (80.0)	35 (45.5)	
Head injury—females			
No head injury	1 (100.0)	25 (75.8)	1.0 (0.04–26.94)
1 or more head injuries	0 (0.0)	8 (24.2)	
Head injury—males			
No head injury	5 (50.0)	20 (71.4)	2.5 (0.57–11.05)
1 or more head injuries	5 (50.0)	8 (28.6)	
Medication^a^			
No analgesics	13 (86.7)	64 (83.1)	0.76 (0.15–3.77)
Analgesics	2 (13.3)	13 (16.9)	

a Data from males and females pooled since no significant differences in frequency were observed.

Statistically significant odds ratios, i.e., with the 95% CI not including 1) are in bold.

Frequencies of head injuries in former football or ice hockey players were similar to other ALS patients (Table 4). The mechanism of head injury was related to these sports only in a single case (zygomatic bone fracture after a head concussion while playing football). Although patients with a positive history of playing football or ice hockey tended to develop first symptoms of ALS earlier than patients without former exposition to these sports, these differences did not reach statistical significance. This was true both for male (52.7 ± 13.4 vs. 57.8 ± 11.9, *F* = 1.44, *p* = .237 ANOVA) and female (59 vs. 62.2 ± 11.0, *F* = 0.008, *p* = .776) football players and male ice hockey players (50.4 ± 15.3 vs. 57.3 ± 11.9, *F* = 1.44, *p* = .237). None of the football players reported exposure to fertilizers, pesticides, herbicides, or solvents.

## 4 Discussion

We investigated the hypothesis that physical activity and different extrinsic risk factors including head injuries predispose for ALS in a cohort of 92 Swiss ALS patients.

### 4.1 The role of head trauma in the development of ALS

It was proposed that (repetitive) head trauma could be a risk factor for developing ALS. An excess occurrence of ALS in Italian football players fuelled such considerations (Beghi, 2013). Different biological mechanisms that may trigger neurodegeneration after head injury include inflammatory and glutamate excitotoxicity (Arundine & Tymianski, 2004) and oxidative stress (Frantseva, Perez Velazquez, Tonkikh, Adamchik, & Carlen, 2002).

Amongst reported head/neck injuries, a history of mTBI (with or without loss of consciousness) was most frequent in our ALS cohort (16.7%). Compared to the lifetime history of traumatic brain injury with confirmed loss of consciousness in the general population in developed nations (12.1%) and the estimated cumulative prevalence of mTBI (with or without loss of consciousness) in Switzerland by age 59.2 years (according to the mean age at ALS‐symptom onset, 13.7%) (SUVA Medical, 2014), a significant excess occurrence of mTBI in our ALS cohort seems unlikely. These findings contrast previous case series (Chen et al., 2007; Pupillo et al., 2012; Schmidt, Kwee, Allen, & Oddone, 2010; Seelen et al., 2014; Strickland et al., 1996) and a meta‐analysis (Chen et al., 2007). However, in two studies excess occurrence was significant only within 1 year after head injury (Peters et al., 2013; Turner et al., 2010), rather suggesting accidents provoked by early signs of ALS than ALS secondary to head trauma (Turner et al., 2010). Furthermore, studies with higher class of evidence (based on medical records rather than patients’ recall) showed no excess of head trauma prior to symptom onset (Armon & Nelson, 2012).

While not increasing the risk for ALS, head injury may accelerate disease progression, resulting in earlier symptomatic presentation. Indeed, in our cohort a history of head/neck injuries was linked to younger age at symptom onset (54.1 vs. 61.8 years, *p* = .013). This, however, contrasts previous observations that a single head trauma is not associated with age at symptom‐onset and the rate of disease progression (Fournier, Gearing, Upadhyayula, Klein, & Glass, 2015). Noteworthy, about half of our patients with a history of head/neck injury reported more than one event and for analysis we pooled patients with one or more head/neck injuries. Therefore, the effect of head trauma on age at disease onset may be augmented by *repetitive* head/neck trauma.

### 4.2 Does vigorous physical activity increase the risk for ALS?

We found no association between a history of vigorous physical activity and age at first symptoms. Therefore, our data do not support that higher levels of premorbid physical activities accelerate the onset of ALS, confirming previous observations (Veldink et al., 2005). The positive correlation between total MET values and age at symptom onset is in line with recent work, suggesting that physical activity may eventually be even protective against motor neuron disease (Pupillo et al., 2014). However, this finding must be interpreted with caution, as disease onset at higher age may have allowed those patients to remain physically active longer, resulting in higher total MET values.

Overall, both rates of patients that considered themselves physically active (95%) and reported vigorous physical activities during their presymptomatic stage (51%) were larger than in the literature: previously, the rate of physically active people was reportedly lower in ALS patients (in the presymptomatic stage) than in the controls (67% vs. 74%, adjusted OR = 0.65, 95%‐CI = 0.48–0.89) in one study (Pupillo et al., 2014). Since we did not obtain a control population, we cannot put this value in relation to the rate in the general Swiss population. Likewise, rates for participants with vigorous physical activities (including marathon and triathlon) were not different for patients and controls (16% vs. 14%, adjusted OR = 1.24, 95%‐CI = 0.96–1.61) in a Dutch study, not supporting an association between vigorous physical activity and the ALS risk (Huisman et al., 2013). The higher rate of 95% of physically active patients obtained here might be related to how this question was phrased in the questionnaire. Noteworthy, we did not provide any requirements that had to be met to qualify for “being physically active” in our questionnaire.

While an increased risk has been reported in Italian football players (Belli & Vanacore, 2005; Chio et al., 2005), American football players (Lehman et al., 2012), and sport‐related physical exercise in general (Beghi et al., 2010), no such link has been found in population‐based case–control studies in Europe (Pupillo et al., 2014) and the US (Longstreth et al., 1998). With several extensive literature reviews reporting no evidence for a link between sports‐ or work‐related physical activity and ALS (Armon, 2007; Hamidou et al., 2014; Veldink et al., 2005), it was proposed that not increased physical activity per se, but rather a—yet unknown—genetic profile or lifestyle‐promoting physical fitness increases ALS susceptibility (Huisman et al., 2013; Turner, Wotton, Talbot, & Goldacre, 2012). This hypothesis was supported by the observed discrepancy between an increased risk for ALS with higher levels of leisure time physical activities and lack of association of ALS risk and occupational physical activity and the absence of a dose–‐response relationship (Huisman et al., 2013). Harwood and colleagues proposed that such a susceptibility could be reflected in a specific physical constitution with a relatively larger fraction of type 1 muscle fibers (Harwood et al., 2009) and Longstreth and colleagues hypothesized that the effect of toxins on motor neurons could be potentiated by vigorous physical activity (Longstreth, Nelson, Koepsell, & van Belle, 1991). With a very high rate of physically active study participants and with a history of vigorous physical activity in half of them, our cohort would fit well the profile of patients being more susceptible to ALS.

### 4.3 Do football and ice hockey predispose to ALS?

We identified vigorous physical activities at a higher rate in former football and ice hockey players than in the other ALS patients (*p* = .022). However, total individual MET values were not dominated by football or ice hockey, i.e., other physical activities substantially contributed to total MET values as well. Furthermore, football and ice hockey players were not amongst the physically most active ALS patients and compared to other ALS patients, the prevalence of head/neck injuries was not higher in former football and ice hockey players. While none of the former football players reported exposure to fertilizers, pesticides/herbicides, or solvents, such exposure may have remained unrecognized by the players. An impact of drugs or dietary supplements was considered as more likely by others (Armon, 2007; Belli & Vanacore, 2005). This includes anabolic steroids, which might increase the risk for ALS via their anabolic or androgen‐like effect (Armon, 2007). Asking specifically about performance‐enhancing drugs, intake was denied by all our patients. The rate of prolonged (>30 days) daily intake of analgesics did not depend on a history of football or ice hockey, suggesting that any potential impact of analgesics on the ALS risk would be independent from former participation in these sports. Noteworthy sample size in our study was probably too small to identify drug‐related effects on ALS.

While former male football players and ice hockey players became symptomatic earlier by on average 5.1 and 6.9 years, respectively, these differences were not significant. Based on a series of 18 former professional Italian male football players diagnosed with ALS, Chio et al. (2005) reported a mean age at symptom onset of 51.2 years (±12.4; 1 *SD*; Chio et al., 2005), which matches the values observed here (52.7 ± 13.4 years) closely. Considering the mean and *SD* of age at symptom onset from an Italian ALS registry taken for comparison (63.4 ± 11.2 years; Piemonte and Valle d'Aosta Register for Amyotrophic Lateral Sclerosis, 2001), mean age at symptom onset in the football players studied by Chio and colleagues is likely not significantly reduced compared to the level of −2 *SD* of the mean (41.0 years). Albeit not significant, such repeatedly observed tendencies for lower average age at symptom‐onset in former football players is remarkable and need further evaluation in future studies. However, the underlying causes for such an accelerated symptom onset remain unclear.

### 4.4 Study limitations

With its retrospective, questionnaire‐based design, the most important limitation is potential recall bias. This bias may affect any conclusions on the frequency of certain events as risk factors for ALS. However, with a focus on sports‐related physical activities and their possible association with ALS, subgroup analyses could be performed within our case series without any restrictions emerging from this potential bias. We did not include an age‐ and gender‐matched control group in this study, which poses certain limitations on the interpretation of our findings, including the frequency of certain sports activities and the representativeness of MET‐values calculated. Facing these restrictions, we focused on patterns within the ALS study sample.

A response rate of approximately 50% imposes the risk for a selection bias in our ALS patient cohort. Reasons for not having returned the questionnaire include death in the same year as registration to the registry used to identify ALS patients and missing German‐language skills (we only had a questionnaire available in German). In our questionnaire 88% indicated German as their mother tongue. Taking in mind that about 30% of Swiss inhabitants having a mother tongue different than German, this likely suggest that there was a bias based on language. However, there is no reason to assume that the physical activity profile or the course of disease in non‐German‐speaking Swiss inhabitants is distinct. Furthermore, both the distribution of gender (male‐to‐female ratio = 1.24:1) and gender‐dependent differences in average age at symptom onset (56.8 vs. 62.1 years) were in line with values from previous ALS populations, reporting male‐to‐female ratios ranging between 0.8:1 and 2.7:1 (Alonso et al., 2009; Cronin et al., 2007; Uenal et al., 2014) and a tendency for earlier symptom‐onset in men than women (Logroscino et al., 2010; Piemonte and Valle d'Aosta Register for Amyotrophic Lateral Sclerosis, 2001; Uenal et al., 2014). The male‐to‐female ratio of former registered football players in our ALS population of 10:1 was identical to the reported ratio of registered players worldwide (FIFA big count, 2006). This suggests that the composition of our ALS population was representative for this disorder as well as for participation in football. Due to the focus of this study on physical activities and sports, physically active/sports inclined patients may have been more willing to participate than average ALS patients. This bears a certain risk that our study overestimates the role of physical activities in ALS.

We instructed patients to focused on the presymptomatic stage when answering questions related to physical activities and potential exposure to possible risk factors. Obviously, there is an asymptomatic period in ALS, potentially bearing the risk that reported exposures occurred only after disease onset. However, the focus of our questionnaire was on physical activities and sports already many years or decades ago. This was also controlled by asking for age at exposure to a certain condition, making it unlikely that these activities were performed after onset of disease.

## 5 Conclusions

Our data suggests that one or several episodes of head trauma may accelerate neurodegeneration in ALS, resulting in earlier clinical manifestation. Underlying mechanisms for such an effect remain unclear, but may be related to microtrauma or oxidative stress of motor neurons. In accordance with recent literature reviews and meta‐analyses, we did not find significant links between vigorous physical activity and earlier ALS disease onset. Furthermore, we did not identify any environmental risk factors (such as physical activity, medication or head injuries) predisposing former football or ice hockey players to ALS. While (repetitive) head traumata may be linked to earlier symptom onset, our observations fit well into the previously stated hypothesis that not increased physical activity per se but other unknown environmental factors and/or an—as yet unknown—genetic profile or lifestyle‐promoting physical fitness increases ALS susceptibility (Huisman et al., 2013).

## Conflicts of Interest

Nina Feddermann‐Demont reports no competing interests. Astrid Junge reports no competing interests. Konrad Weber reports no competing interests. Michael Weller reports no competing interests. Jiri Dvorak reports no competing interests. Alexander Tarnutzer reports no competing interests.

## Authors Contributions

Dr. Feddermann‐Demont conceived of the study, designed the questionnaire, participated in the data analysis, interpreted the data, was involved in drafting of the manuscript, critically revised the manuscript for content and approved the final version. Prof. Junge conceived the study, designed the questionnaire, participated in the data analysis, interpreted the data, critically revised the manuscript for content and approved the final version. Prof. Weller was involved in the study design and preparation, critically revised the manuscript for content and approved the final version. Dr. Weber participated in the data analysis and interpretation, critically revised the manuscript for content and approved the final version. Prof. Dvorak was involved in the study design and preparation, critically revised the manuscript for content and approved the final version. Dr. Tarnutzer was involved in the study design, participated in the data analysis, interpreted the data, drafted and critically revised the manuscript for content and approved the final version. Statistical analysis was conducted by Dr. Tarnutzer.
