# Supplementary material for: Prevalence of potential sports‐associated risk factors in Swiss amyotrophic lateral sclerosis patients
Source: Brain Behav. 2017 Mar 16;7(4):e00630. doi: 10.1002/brb3.630 (PMC5390828; doi:10.1002/brb3.630)
Supplement: Supplementary file 1 [file BRB3-7-e00630-s001.pdf]

Supplementary file 1: additional e-tables

e-Table 1: *Drug intake over a period of at least one month on a daily basis before symptom onset*

*type of drug**	patients	%
analgesics	15	16,3
both NSAIDs† and OPKs‡	4	4,3
NSAIDs only	7	7,6
OPKs only	4	4,3
other drugs§	5	5,4
any substance	19	20,7
number of analgesics taken (NSAIDs, OPKs)	patients	%
no	77	82,6
1 drug	10	10,9
2 drugs	1	1,1
3 drugs	3	3,3
4 drugs	1	1,1

* Intake of several substance classes in single patients possible.

† NSAIDs=Nonsteroidal anti-inflammatory drugs (diclofenac (n=6), celecoxib (n=1), ibuprofen (n=3), mefenamid (n=1), rofecoxib (n=1), azapropazone (1)).

‡ OPK= Other analgesics (paracetamol (n=6), paracetamol plus coffein (n=1), metamizol (n=2), tramadol (n=2))

§ Other drugs (omeprazol (n=2), hydroxichloroquin (n=1), temazepam (n=1), lamotrigin (n=1), cortison (n=1), diazepam (n=1), acetylsalicysäure (n=1), tamsulosin (n=1), enalapril (n=1)

e-Table 2: *average MET value (in millions) of highest activity of ALS patients playing FB and/or IH and non FB/IH players*

**e-Table 2:** *Average MET value (x 10^6) of highest activity in former football/ice hockey players vs. non-players*			
**Sports**	**total (mean±1SD)***	**highest activity of all activities (mean±1SD)†**	**activity due to IH or FB if present (mean±1SD)**
neither IH nor FB	13.9 (13.2)	9.6 (11.0)	N/A
football only	13.9 (12.4)	8.1 (5.9)	4.7 (5.5)
ice hockey only	18.1 (11.9)	14.2 (7.2)	0.7 (1.0)
both	5.8 (4.5)	5.0 (5.3)	5.0 (5.3)
**all**	**13.9 (12.9)**	**9.5 (10.3)**	**9.5 (10.3)**

Abbreviations: FB=football; IH=ice hockey

* F=0.361, p=0.781 (ANOVA)
† F=0.393, p=0.759 (ANOVA)
